# Golgi_DF: Golgi proteins classification with deep forest

**DOI:** 10.3389/fnins.2023.1197824

**Published:** 2023-05-12

**Authors:** Wenzheng Bao, Yujian Gu, Baitong Chen, Huiping Yu

**Affiliations:** ^1^School of Information Engineering, Xuzhou University of Technology, Xuzhou, China; ^2^Department of Stomatology, Xuzhou First People’s Hospital, Xuzhou, China; ^3^The Affiliated Hospital of China University of Mining and Technology, Xuzhou, China; ^4^Department of Neurosurgery, The Hospital of Joint Logistic, Quanzhou, China

**Keywords:** Golgi proteins, fusion features, deep forest, UniRep, PHATE, light GBM

## Abstract

**Introduction:**

Golgi is one of the components of the inner membrane system in eukaryotic cells. Its main function is to send the proteins involved in the synthesis of endoplasmic reticulum to specific parts of cells or secrete them outside cells. It can be seen that Golgi is an important organelle for eukaryotic cells to synthesize proteins. Golgi disorders can cause various neurodegenerative and genetic diseases, and the accurate classification of Golgi proteins is helpful to develop corresponding therapeutic drugs.

**Methods:**

This paper proposed a novel Golgi proteins classification method, which is Golgi_DF with the deep forest algorithm. Firstly, the classified proteins method can be converted the vector features containing various information. Secondly, the synthetic minority oversampling technique (SMOTE) is utilized to deal with the classified samples. Next, the Light GBM method is utilized to feature reduction. Meanwhile, the features can be utilized in the penultimate dense layer. Therefore, the reconstructed features can be classified with the deep forest algorithm.

**Results:**

In Golgi_DF, this method can be utilized to select the important features and identify Golgi proteins. Experiments show that the well-performance than the other art-of-the state methods. Golgi_DF as a standalone tools, all its source codes publicly available at https://github.com/baowz12345/golgiDF.

**Discussion:**

Golgi_DF employed reconstructed feature to classify the Golgi proteins. Such method may achieve more available features among the UniRep features.

## 1. Introduction

Golgi is an essential organelle in eukaryotic cells (Yang et al., 2019). Its main function is to store, package, and classify proteins. Golgi proteins are mainly composed of Cis-Golgi proteins and trans-Golgi proteins (Su et al., 2022). The main task of Cis-Golgi is to accept and process at the same time. The main task of trans-Golgi is to release proteins labeled and processed by vesicles. Studies have shown that dysfunction of the Golgi apparatus in cells can cause diseases such as diabetes (Wang and Zou, 2023), Parkinson’s disease (Gonatas et al., 1998), Alzheimer’s disease (Gonatas et al., 1998), and some cancers. The current treatment methods can only partially cure the disease (Elsberry and Rise, 1998), which is challenging to meet the needs.

With the development of machine learning technology, machine learning model has been applied to the related research of protein analysis (Villeneuve et al., 2017; Wei et al., 2017a, 2019; Zeng et al., 2018; Hou et al., 2019; Yuan et al., 2019; Hummer et al., 2020). However, there are few studies on the classification of Golgi protein types, and only a few are used to study the resident proteins of Golgi. In the past few years, Van Dijk et al. (2008) proposed a method to predict the type of type II membrane protein. It utilized a linear kernel support vector machine as a classifier. Ding et al. (2011) Utilized PSEAAC and customized Markov discriminator to identify Golgi protein types with an accuracy of 74.7%. Then, the improved interval dipeptide combination method enhances the accuracy and realizes the prediction accuracy of 85.4% (Ding et al., 2013). Jiao and Du (2016a) utilized the position-specific physicochemical properties (PSPCP) of amino acid residues to extract features and improved the model’s prediction accuracy to 86.9%. After that, they combined PSPCP with Chou’s pseudo amino acid composition Jiao and Du (2016b). Lv et al. (2019) designed a random forest sub-Golgi protein classifier Rfgpt, which utilized 2-gap dipeptide and split amino acid composition as feature vectors, and combined with synthetic minority oversampling technique (SMOTE) and analysis of variance (ANOVA) feature selection method, and the prediction accuracy is 90.5%.

**Figure 1:**
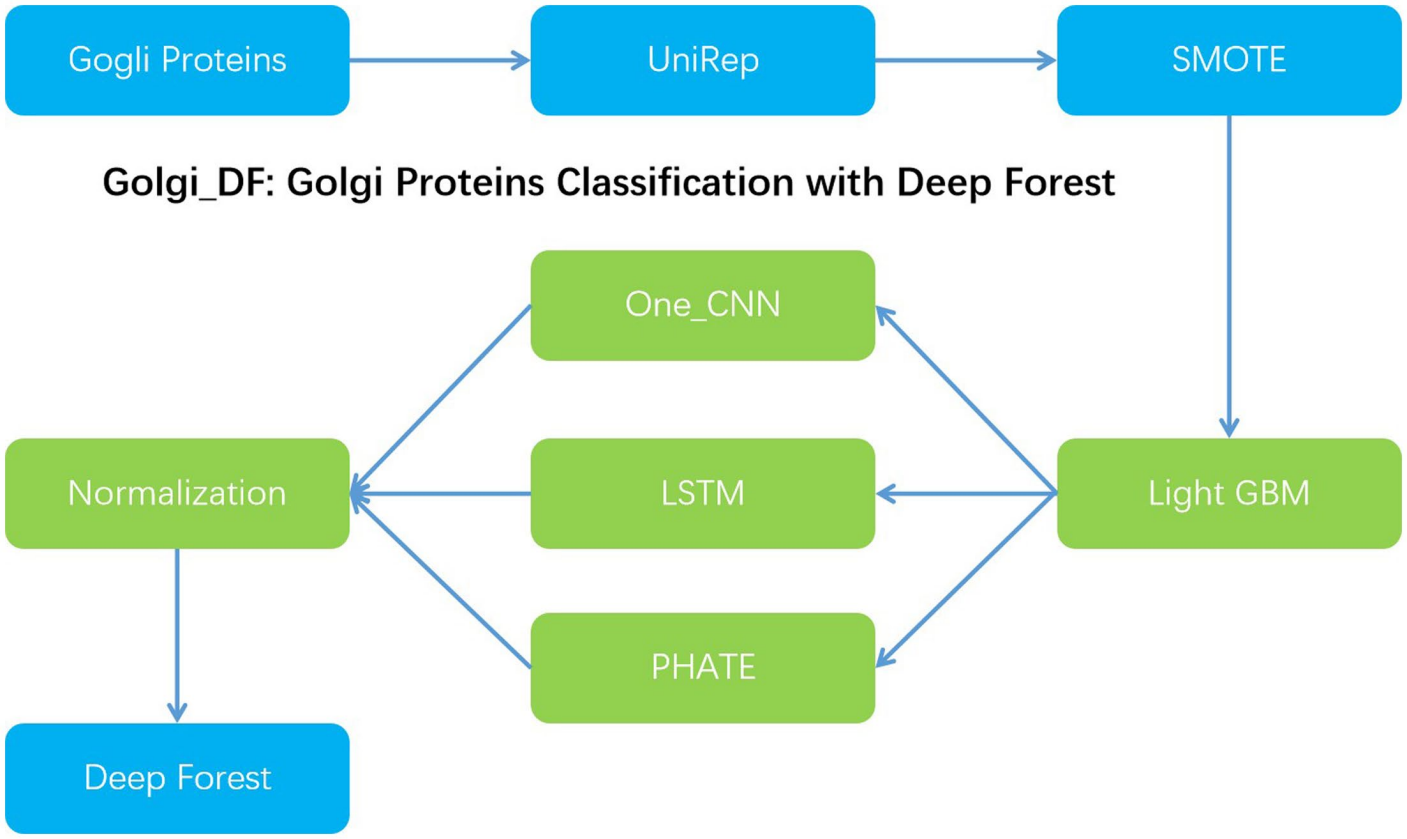
Work flow chart of Golgi_DF: Golgi proteins classification with deep forest.

In order to improve the classification effect of Golgi resident proteins, we proposed Gogli_DF model to classify the Golgi proteins with the deep forests model. Firstly, we utilized the UniRep method to achieve 1900-dimensional vector features. Secondly, we utilized the synthetic minority oversampling Technology (SMOTE) to deal with the imbalance issue of the classified samples and then used the light gradient boosting machine(Light GBM) method to reduce the dimension of the feature vector to 200-dimensional. Nextly, one-dimensional convolution, multi-layer LSTM, and PHATE dimensionality reduction are employed to extract the feature information, respectively. At the same time, the same 32-dimensional dense layer is used in the penultimate layer of the three models to ensure the consistency of the dimensions of various extracted vectors, which is convenient for the combination and selection of the features extracted by different models. At the same time, normalized normalization is used to preserve the distribution and eliminate the influence of dimension. Then, these feature vectors are spliced horizontally to achieve the purpose of feature fusion. Next, the above-mentioned three features can be employed in the deep forest classification model. With the 5-fold cross validation, the performance can reach 96.3% in Acc, 93.8% in Sn and 96.9% in Sp, respectively (Figure 1).

## 2. Materials and methods

### 2.1. Dataset

The benchmark data set of this experiment comes from the data set constructed by Yang et al. (2019). The data set contains 304 amino acid sequences of Golgi proteins, including 87 positive samples and 217 negative samples. To avoid overfitting, we use 64 Golgi protein amino acid sequences that are fixed and not included in the training set. The selected divided test set contains 64 Golgi protein amino acid sequences with a ratio of positive and negative samples of about 1:4, including 13 positive samples and 51 negative samples. The feature extraction of initial data is an essential step in classification. Choosing an appropriate feature extraction method will significantly enrich the information to provide an information guarantee for improving classification accuracy.

### 2.2. UniRep feature

UniRep can be treated as a feature extraction method trained based on 24 million uniref50 primary amino acid sequences. The feature is trained to minimize the loss of cross-entropy in the prediction of the next amino acid. Therefore, we can learn all kinds of information about the sequence to ensure the richness of information and complete the unification of vector length. The final feature is represented by a 1900-dimensional fixed-length vector.

### 2.3. Light GBM

Light GBM algorithm is an improvement of the traditional GBM algorithm, which reduces the memory consumption and calculation cost through the histogram algorithm. At the same time, the leaf-wise strategy with depth limit is used to replace the level-wise decision tree growth strategy used by the traditional GBM tool. Another optimization of light GBM is the acceleration of histogram difference, which improves the speed. In this paper, light GBM can extract the 200-dimensional features from 1900-dimensional ones.

### 2.4. Smote

According to the positive and negative samples’ unbalanced issue, we need to use resampling to solve this problem. The SMOTE method proposed by Chawla et al. (2002). Such a method is a method of random undersampling for large samples and random oversampling for small samples. This algorithm is a standard method to deal with unbalanced data (Blagus and Lusa, 2013; Cateni et al., 2014; Díez-Pastor et al., 2015; Sáez et al., 2015; Nath and Subbiah, 2016; Ma and Fan, 2017; Wang et al., 2019).

The detailed steps is as follows:

1. Set the multiplier for up sampling to *N*.
2. Find K-nearest neighbor of sample $x_{i}$ from the sample of interface residues, represented by $x_{i\left(n\right)}$, n∈{1,…,k}, and randomly select n samples, represented by $y_{1},\ldots ,y_{N}$.
3. Synthesize new samples $x_{i1}$,

(1) $$x_{i1}=x_{i}+\xi_{1}\left(y_{1}-x_{i}\right)$$

Among them, $\xi_{1}$ is a random number in (0, 1). Repeat the above process n times until we get new samples: $x_{i\left(\mathrm{ne}\right)}$, ne∈{1,…,N}.

These newly synthesized samples are added to the original samples to form a new and more balanced data set.

### 2.5. Multi-layer LSTM and one-dimensional CNN

**Figure 2:**
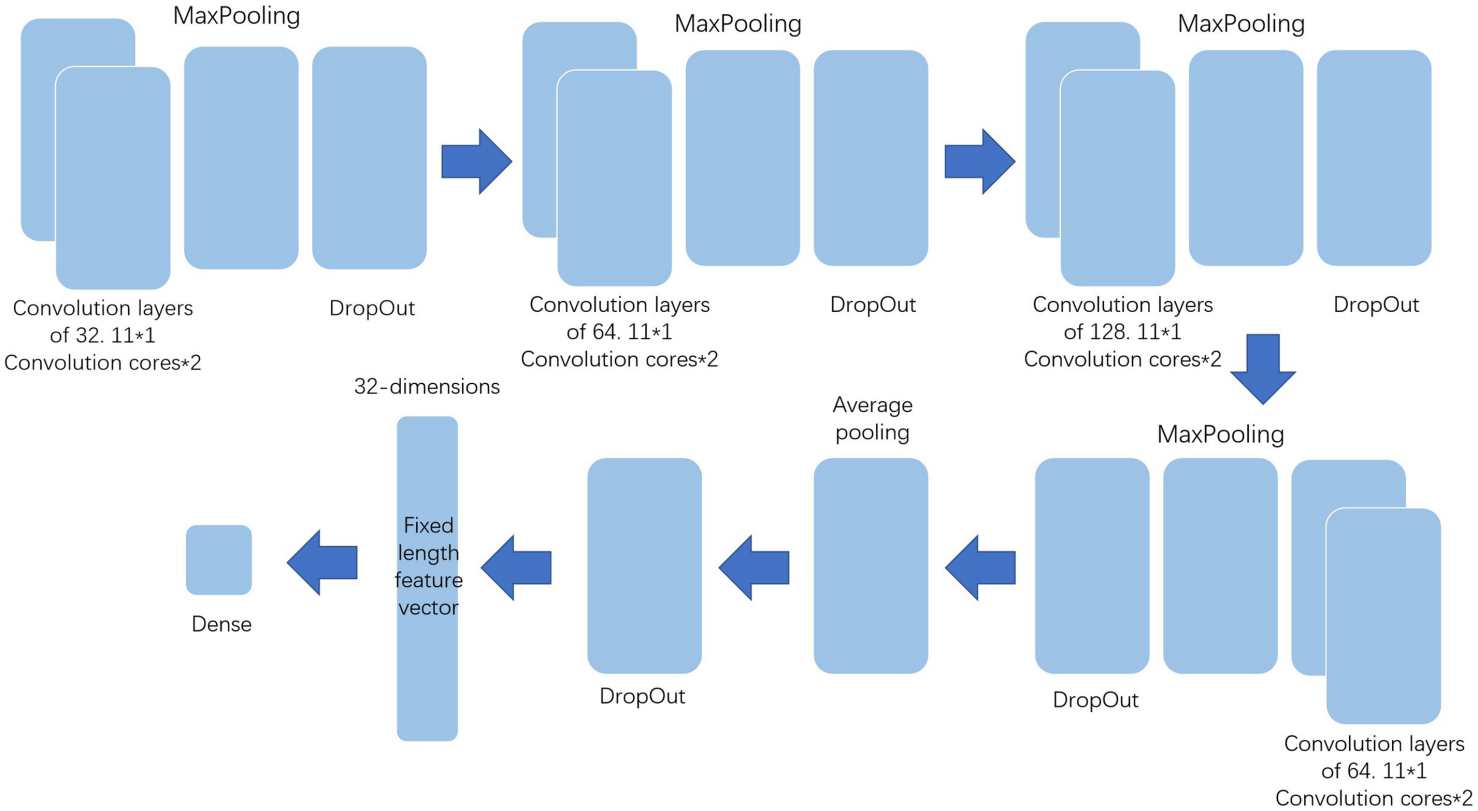
One-dimensional Convolution.

**Figure 3:**
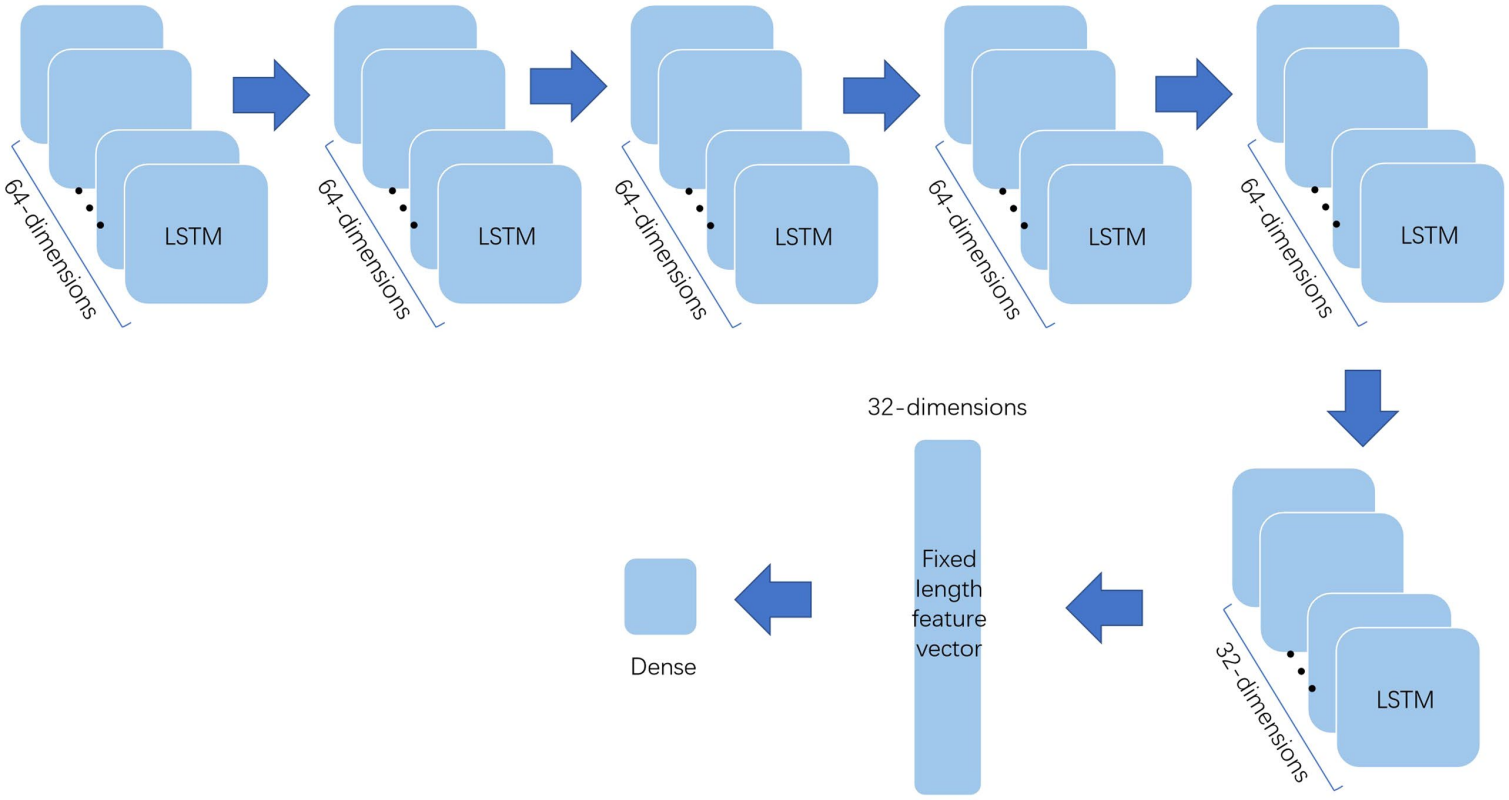
Multi-Layer LSTM.

The processed data should be further extracted by various methods before deep forest to improve the classification effect. This paper uses multi-layer LSTM and one-dimensional convolution to extract the features and the structure of them show in Figures 2, 3. Among them, the multi-layer LSTM comprises five 64-dimensional LSTM layers, one 32-dimensional LSTM layer, and two density layers in sequence. Among the last two density layers, the first density layer is 32 dimensional, also the data source of the previous feature extraction. It can ensure that the final extracted information is a 32-dimensional fixed-length feature vector. At the same time, the second density layer is 1-dimensional, which is convenient for comparison with the label, to back-propagate the correction parameters to force it to express the corresponding features. The one-dimensional convolution consists of 20 layers in sequence. The first 16 layers are, respectively, composed of two convolution layers of 32 11 * 1 convolution cores, one max-pooling layer, one dropout layer, two convolution layers of 64 11 * 1 convolution cores, one max-pooling layer, one dropout layer, two convolution layers of 128 11 * 1 convolution cores, one max-pooling layer, one dropout layer, and the convolution layers of the last two 64 11 * 1 convolution cores, one max-pooling layer, A dropout layer. The last four layers are the average pooling layer and dropout layer, plus two density layers, the same as multi-layer LSTM. The first-density layer is also 32-dimensional as the data source for the final feature extraction, while the second-density layer is 1-dimesional.

### 2.6. PHATE

At the same time, this paper uses PHATE dimension reduction as the feature extraction method. Unlike the UniRep used in the transformation process from sequence to feature vector, which needs to retain enough original information, PHATE focuses on preserving the local relationship between data points and learning overall spatial features, providing a new feature analysis angle for the classification of the deep forest. The dimension of the eigenvector is reduced to 32-dimensional, which is consistent with other methods. PHATE is a nonlinear and unsupervised method that combines the advantages of PCA and tsne, retains the local and global relationship between data, and accurately reflects the high-dimensional data set discussed.

The detailed operations are as follows:

1. The value of the eigenvector of each sequence is expressed as $x_{n},n\in \left\{1,\ldots \mathrm{,k}\right\}$, k equals to 200, that is, the dimension of the eigenvector. Gaussian kernel function is used to quantify the similarity between $x_{a}$ and $x_{b}$, a,b∈{1,…,k}, according to the Euclidean distance between them. The expressed in Gaussian kernel function is $k_{z}\left(x_{a}{\mathrm{,x}}_{b}\right)$,


(2) $$k_{z}\left(x_{a}{\mathrm{,x}}_{b}\right)=\exp\left(-{\left\|x_{a}-x_{b}\right\|}^{2}/\varepsilon \right)$$

Where ε is the bandwidth measurement, which is used to determine the neighborhood radius captured by the kernel function.2. The Markov random walk diffusion process is used to diffuse in the data. The initial probability of random walk is $P_{\varepsilon },$


(3) $$P_{\varepsilon }=\frac{k_{\varepsilon }\left(x,y\right)}{v_{\varepsilon }\left(x\right)}$$

where


(4) $$v_{\varepsilon }\left(x\right)=\underset{z\in x}{\sum }k_{\varepsilon }\left(x,z\right)$$

Thus, the transition probability matrix of a single time step from sequence to sequence can be calculated, and the probability matrix can be improved to the best step to learn the global structure of the data.

3. Calculate the potential distance $ℜ\left(x_{a}{\mathrm{,x}}_{b}\right)$,


(5) $$ℜ\left(x_{a}{\mathrm{,x}}_{b}\right)={\left\|U_{x_{a}}^{t}-U_{x_{b}}^{t}\right\|}_{2}$$

where


(6) $$U_{x_{a}}^{t}=-\log\left(p_{x_{a}}^{t}\right)$$

Where $p_{x_{a}}^{t}$ is $x_{a}$’s corresponding transition probability.

4. Use metric multidimensional scaling (MDS metrics) by minimizing $stress\left({\hat{x}}_{1},\ldots ,{\hat{x}}_{32}\right)$,


(7) $$stress\left(\hat{x},\ldots ,{\hat{x}}_{32}\right)=\sqrt{\underset{i,j}{\sum }{\left({ℜ}_{(x_{i},x_{j)}}^{t}-{\left\|\overset{\wedge }{x_{i}}-\overset{\wedge }{x_{j}}\right\|}_{J}\right)}^{2}/\underset{i,j}{\sum }{\left({ℜ}_{(x_{i},x_{j)}}^{t}\right)}^{2}}$$

So far, the data has been captured in the MDS embedding, and a fixed length vector with a length of 32 has been obtained.

### 2.7. Feature fusion

Using multi-layer LSTM, one-dimensional convolution network, and PHATE dimensionality reduction method to extract features, three groups of data with each sequence corresponding to a 32-dimensional fixed length vector are obtained. These vectors are spliced horizontally, and each sequence obtains a 96-dimensional fixed-length vector, which is normalized by the normalized method. While scaling to between 0 and 1, the distribution of the original data is retained, avoiding the influence of the dimension of the feature vectors extracted by different classifiers on the classification results.

### 2.8. Deep forest

**Figure 4:**
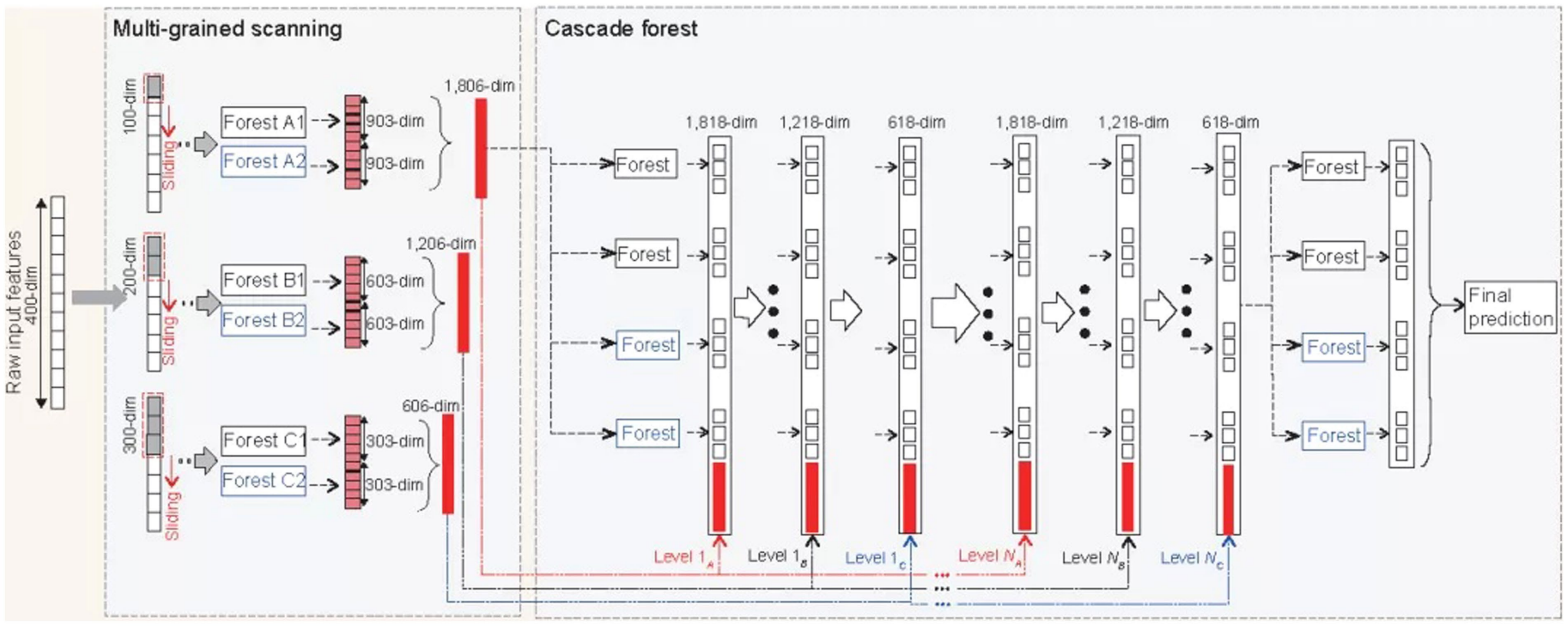
Deep forest.

This paper uses the deep forest as the bottom classifier. The deep forest was proposed by Zhou and Feng (2019) They find that when the differences in learning samples are fully reflected, the effect of integrated learning will be improved accordingly. The deep forest is an integration of traditional forests in breadth and depth. This classifier uses a new decision tree integration method, a forest, and a cascade structure to make the forest do representation learning. The advantage of the classifier is that it can process data of different scales and has a more stable and good learning performance. The traditional deep neural network needs large-scale training data, and the forest works as usual when there is only small-scale training data. Because the data scale of this paper is small, and as a primary classifier, its high stability also provides an essential guarantee for the performance of classification, so the deep forest is used as the primary classifier of this paper, and the detailed steps of this algorithm demonstrated in Figure 4 (Zhou and Feng, 2019).

### 2.9. Evaluation performances

In the classification of Golgi resident proteins, it is an essential step to select appropriate evaluation indexes to evaluate the performance of the model. Its positive and negative samples represent CIS and trans-Golgi proteins, respectively. In this experiment, accuracy (ACC), AUC (area under ROC curve), F1–score, sensitivity (SN), specificity (SP), and Matthews correlation coefficient (MCC) are utilized in this work (Pedregosa et al., 2011; Wei et al., 2017b,c; Zeng et al., 2017; Hu et al., 2018; Song et al., 2018; Lin et al., 2019; Zhang et al., 2019). The calculation method is as follows:


(8) $$ACC=\frac{TP+TN}{TP+FN+TN+FP}$$

(9) $$F1-score=\frac{2\times TP}{2\times TP+FN+FP}$$

(10) $$Sn=\frac{TP}{TP+FN}$$

(11) $$Sp=\frac{TN}{TN+FP}$$

(12) $$MCC=\frac{TP\times TN-FP\times FN}{\sqrt{\left(TP+FP\right)\times \left(TP+FN\right)\times \left(TN+FN\right)\times \left(TN+FP\right)}}$$

The above-mentioned parameters, including TP, TN, FP, and FN, mean the sample labels and the sample calculated labels.

## 3. Discussions and results

To prove that the combination of multi-layer LSTM and one-dimensional convolution network is effective for the deep forest, the feature extraction method is extracted by the machine learning method, and the model’s efficiency is explained. Therefore, these feature extraction methods are connected with deep forests, and the prediction accuracy is compared with the effect of connecting other models with deep forests. Specifically, RESNET, multi-layer CNN, random forest, and elastic net are used to compare with multi-layer LSTM network and one-dimensional convolution network. Table 1 shows the comparison results on the evaluation indexes ACC, MCC, F1 score, AUC, Sn, and SP after connecting the deep forest with the six machine learning models as the means of feature extraction and the matrix spliced with the feature vectors extracted from multi-layer LSTM network and one-dimensional convolution network as the feature extraction results. In the machine learning model for feature extraction, 14-layer CNN consists of one convolution layer of 16 3 * 3 convolution cores, one batch normalization layer, one max-pooling layer, one convolution layer of 32 3 * 3 convolution cores, one batch normalization layer, one max-pooling layer, one drop out layer, one convolution layers of 64 3 * 3 convolution cores, one batch normalization layer, one max-pooling layer and one drop out layer, In addition, it is composed of a global average pooling layer, a 32-dimensional density layer, and a 1-dimensional density layer. Of the last two density layers, the first density layer is 32-dimensional, also the data source of the last feature extraction, while the second-density layer is 1-dimensional. The last two density layers and the same idea of feature extraction and weight correction are adopted in the later RESNET, multi-layer LSTM, and one-dimensional convolution models. In RESNET, a data import part is composed of a convolution layer, batch normalization layer, activation layer, and pooling layer of 3 * 3 convolution kernel, a residual part composed of four residual blocks composed of 64, 128, 256, 512 filters, plus the global pooling layer and two 32-dimensional and 1-dimensional density layers as above. Because the results of some classifiers fluctuate greatly, this paper runs each classifier many times, takes the result with the highest accuracy in each time as the running result of that time, and runs 10 times to take the average value as the final result of the model.

**Table 1:** Comparison of several machine learning feature extraction.

Model	ACC	AUC	f1-score	Sn	Sp	MCC
ResNet	0.764	0.643	0.414	0.438	0.847	0.281
CNN	0.797	0.701	0.519	0.538	0.863	0.390
RF	0.905	0.891	0.787	0.869	0.914	0.732
ElasticNet	0.914	0.889	0.800	0.846	0.931	0.747
LSTM	0.925	0.910	0.826	0.885	0.935	0.782
1_Dim_Conv	0.934	0.910	0.841	0.869	0.951	0.802
LSTM+1_Dim_Conv	0.947	0.938	0.875	0.923	0.953	0.844

It can be seen from the results that on ACC and MCC, it can be found that both multi-layer LSTM and one-dimensional convolution networks are ahead of other models. After combination, the ACC value is increased by 0.031 compared with multi-layer LSTM and 0.013 compared with a one-dimensional convolution network. At the same time, the score of the combination of multi-layer LSTM and one-dimensional convolution network is the highest on the F1 score, which not only exceeds other machine learning methods but also improves by 0.075 and 0.034 respectively, compared with multi-layer LSTM and one-dimensional convolution network, indicating the robustness of the model. On the evaluation index AUC, the combined feature extraction model is also excellent, which is 0.028 and 0.028 higher than the best multi-layer LSTM and one-dimensional convolution network, respectively, indicating that the combined model has the best generalization performance than other models. The above results show that the stitching feature can improve the model’s classification performance.

In addition, this paper also compares convolution with different structures and LSTM classifiers. As above, we run 10 times and take the average value as the final result of the model. For CNN, this paper compares the 7-layer CNN composed of a 1-layer 16-dimensional convolution, a batch normalization layer, a max-pooling layer, a dropout layer, a global average pooling layer, a 32-dimensional density layer, and a 1-dimensional density layer, and the convolution layer composed of two 16 3 * 3 convolution cores, a batch normalization layer, a max-pooling layer, two 32 3 * 3 convolution cores, and a batch normalization layer, A multi-layer CNN network composed of one max-pooling layer, two convolution layers of 64 3 * 3 convolution cores, one batch normalization layer, one max-pooling layer, plus a global average pooling layer, a 32-dimensional sense layer, and a 1-dimensional sense layer is compared with the 14 layer CNN network previously used. It is found that no CNN of any structure has achieved an available feature extraction effect. Nevertheless, Compared with other CNN networks, the above 14-layer CNN model has achieved better results. Firstly, it shows that the appropriate number of network layers in the CNN network has a specific impact on the results. Secondly, the above 14-layer CNN network can be considered as representative and reference significance; For LSTM, this paper compares the three-layer LSTM network with a single LSTM layer as the feature extraction, and achieves similar results, indicating that increasing the number of layers in Golgi protein classification does not bring ideal results; Finally, for one-dimensional convolution, the 10 layers one-dimensional convolution model is compared. This model consists of a convolution layer of 32 11 * 1 convolution cores, a max-pooling layer, a dropout layer, and a convolution layer of 64 11 * 1 convolution cores, a max-pooling layer, a dropout layer, an average pooling layer, and a dropout layer, plus two sense layers. The first sense layer is also 32 dimensions as the final feature extraction data source, and The second density layer is 1D. It is found that the multi-layer one-dimensional convolution model used in this paper has a better effect.

This paper also uses different dimensionality reduction methods to extract the information of the original data, which are reduced to 32 dimensions, the same as the dimension removed by the classifier before and then directly put into the deep forest classifier for classification. This paper attempts PCA, Lasso, linear regression, PHATE, and ridge ones. These dimensional reduction methods are standard data analysis methods commonly used for dimensional reduction of high-dimensional data and can be used to extract the main feature components of data. To maintain the consistency of the conditions for obtaining the results, each classifier is run several times. The result with the highest accuracy each time is taken as the running result of the current time. It is consistent with the information extracted by the above classifier, and it is run 10 times to take the average value as the final result of the model.

These experiments found that PHATE and ridge regression dimensional reduction have excellent extraction ability for sequence information. Based on the stitching feature information extracted by LSTM and one-dimensional convolution, this paper adopts the same horizontal stitching as above and then further stitches the feature data extracted by PHATE reduction and ridge regression reduction in the same way. To verify the stability of the model, each classifier is run several times, and the result with the highest accuracy each time is taken as the running result of the current time. Due to the increase in data and the relatively stable classification result, the average value is taken as the final result of the model after running five times. Table 2 compares our last model and other models in the evaluation indexes ACC, MCC, F1 score, AUC, Sn, and Sp. For the features extracted from multi-layer LSTM, one-dimensional convolution, and PHATE, after normalization by normalize method, the extraction effect is best using the information fusion method described above, and its ACC value reaches 0.963, while MCC is improved by 0.043. The AUC value of PHATE can reach 0.959, which has excellent generalization performance. Moreover, when comparing the results of LSTM + one-dimensional convolution without PHATE, the ACC value and AUC value is increased by 0.016, proving the effectiveness of PHATE fusion. At the same time, it is also found that the performance of ridge regression decreases after stitching with the extracted features of the two classifiers, which interferes with the classification of the deep forest by the underlying primary classifier (Table 3).

**Table 2:** Comparison of machine learning feature extraction of different structures.

Model	ACC	AUC	f1-score	Sn	Sp	MCC
7-layer CNN	0.766	0.612	0.366	0.354	0.871	0.238
14-layer CNN	0.797	0.701	0.519	0.538	0.863	0.390
Multi-layer CNN	0.792	0.612	0.356	0.308	0.916	0.268
3-layer LSTM	0.925	0.910	0.826	0.885	0.935	0.782
Multi-layer LSTM	0.916	0.890	0.800	0.846	0.933	0.751
10-layer 1_Dim_Conv	0.894	0.836	0.733	0.738	0.933	0.672
Multi-layer 1_Dim_C	0.934	0.910	0.841	0.869	0.951	0.802

**Table 3:** Comparison of several regression feature extraction and feature fusion.

Model	ACC	AUC	f1-score	Sn	Sp	MCC
LASSO	0.883	0.861	0.740	0.823	0.898	0.671
PCA	0.900	0.874	0.771	0.831	0.918	0.711
LR	0.920	0.893	0.812	0.846	0.939	0.762
PHATE	0.953	0.959	0.894	0.969	0.949	0.868
Ridge	0.953	0.953	0.893	0.954	0.953	0.865
LSTM+1_Dim_Conv	0.947	0.938	0.875	0.923	0.953	0.844
Model A	0.953	0.936	0.885	0.908	0.965	0.858
Proposed method	0.963	0.954	0.909	0.938	0.969	0.887
Model B	0.959	0.957	0.904	0.954	0.961	0.881

Among them, the model A is the feature extraction combination of multi-layer LSTM, one-dimensional convolution, and ridge regression, the Proposed method is the feature extraction combination of multi-layer LSTM, one-dimensional convolution, and PHATE, which is also the final model, and Model B is the feature extraction combination of multi-layer LSTM, one-dimensional convolution, PHATE, and ridge regression.

## 4. Conclusion

This work, the Gogli_DF model has been proposed to classify the Golgi proteins with the deep forests model. Firstly, the UniRep method to achieve 1900-dimensional vector features. Secondly, the SMOTE is employed to deal with the imbalance issue. And then several reconstruction feature methods include Light GBM, one-dimensional convolution, multi-layer LSTM, and PHATE. With the reconstructed features, the deep forest algorithm can be employed as the classification model in this work. With this classification model proposed, several issues can be taken into account.

With the development of big data technology and bioinformatics, the number of available protein sequences has increased significantly. However, due to the complex composition of proteins, it is not easy to classify protein sequences correctly with some traditional methods. Therefore, using machine learning to classify proteins has excellent advantages, and some dimensionality reduction methods of cell sequences can also improve the effect of machine learning model. Firstly, through the pre-training network UniRep method and light GBM dimensionality reduction SMOTE method, this paper unifies the sequences of different lengths into fixed-length feature vectors with relatively fewer features, fully retains various feature information, and solves the problem of the unbalanced classification issue. Through the feature fusion of the multiple information extracted by the machine learning model, including one-dimensional convolution, multi-layer LSTM network, and dimension reduction method PHATE, taking into account the influence of dimension and maintaining the original distribution, we fully mine various information and finally use the deep forest for the final classification. The experimental results show that this method has an excellent performance in the classification of Cis-Golgi proteins and trans-Golgi proteins. At the same time, it is found that the appropriate feature stitching method is helpful to improve the performance, while the effect of feature extraction of some models is good. Still, the performance decreases when combined with other models. Meanwhile, when stitching features, we can consider not only the machine learning model but also the integration of appropriate biological dimensionality reduction methods. The machine learning model can also help improve performance. In the future, in addition to studying better fusion methods, this method and idea can become a powerful tool for bioinformatics and protein research.

## Data availability statement

Publicly available datasets were analyzed in this study. This data can be found at: https://github.com/baowz12345/golgiDF.

## Author contributions

WB and YG can be treated as the co-first authors. WB conceived the method. YG designed the method. BC designed the website of this algorithm. WB and BC conducted the experiments. WB and HY wrote the main manuscript text. All authors contributed to the article and approved the submitted version.

## Funding

This work was supported by the National Natural Science Foundation of China (Grant No. 61902337), Xuzhou Science and Technology Plan Project (KC21047), Jiangsu Provincial Natural Science Foundation (No. SBK2019040953), Natural Science Fund for Colleges and Universities in Jiangsu Province (No. 19KJB520016) and Young Talents of Science and Technology in Jiangsu and ghfund202302026465, Qing Lan Project in Jiangsu, and Qingmiao project of Xuzhou first People’s Hospital.

## Conflict of interest

The authors declare that the research was conducted in the absence of any commercial or financial relationships that could be construed as a potential conflict of interest.

## Publisher’s note

All claims expressed in this article are solely those of the authors and do not necessarily represent those of their affiliated organizations, or those of the publisher, the editors and the reviewers. Any product that may be evaluated in this article, or claim that may be made by its manufacturer, is not guaranteed or endorsed by the publisher.
